# Clinical investigation into the initial diagnosis and treatment of 1,168 lung cancer patients

**DOI:** 10.3892/ol.2014.2777

**Published:** 2014-12-08

**Authors:** QIAN SHAO, JIANBIN LI, FENGXIANG LI, SUZHEN WANG, WEI WANG, SHANSHAN LIU, YINGJIE ZHANG

**Affiliations:** Department of Radiation Oncology, Shandong Cancer Hospital and Institute, Jinan, Shandong 250117, P.R. China

**Keywords:** lung cancer, diagnosis, treatment, prognosis, clinical analysis

## Abstract

The aim of the present study was to analyse clinical data obtained from lung cancer patients, including the initial clinical symptoms upon diagnosis, duration of patient delay in presenting to a doctor, lung cancer stage, treatment strategy and prognosis. A retrospective analysis was conducted of the clinical features of 1,168 lung cancer patients who were initially diagnosed and treated at the Tumor Hospital of Shandong Province (Jinan, China) in 2009. The Kaplan-Meier method and multivariate Cox regression analysis were performed to analyse the influence of gender, age, predominant symptoms, histopathological or cytological type and clinical staging on the overall patient survival. The follow-up rate of the present study was 92.4%, and the 1-, 2- and 3-year survival rates were 80.4, 44.9 and 15.8%, respectively. Multivariate analysis demonstrated that the patient age, extent of the tumour (T stage), extent of lymph node spread (N stage), overall clinical stage and treatment strategy were independent risk factors associated with patient survival. The present study identified that the initial symptoms of lung cancer varied, patient delay was long, the lung cancer cases were diagnosed in late clinical stages and the prognosis was poor.

## Introduction

Lung cancer is the most common type of malignant tumour worldwide, and the morbidity and mortality rates for lung cancer are the highest among malignant tumours (1,2). Lung cancer may remain asymptomatic and lacks common early symptoms. Therefore, it is typically discovered late and diagnosed at an advanced stage; early diagnosis accounts for just 15–25% of lung cancer cases (3). Despite the application of various treatment strategies, the 5-year survival rate for lung cancer patients remains low at <15% (4). In the present study, a retrospective analysis was performed on clinical data from 1,168 lung cancer patients who had received their initial diagnosis and treatment at the Tumor Hospital of Shandong Province (Jinan, China) between January 1 2009 and December 31 2009. The current retrospective analysis reviewed the initial clinical symptoms upon diagnosis, duration of patient delay in presenting to a doctor, lung cancer stage, treatment strategy and prognosis. The aim was to identify factors which influence survival time.

## Patients and methods

### Inclusion criteria

Patients were included on the basis that primary pulmonary lesions were histopathologically or cytologically diagnosed, and the extent and clinical stage of the cancer were determined. Cancer staging was achieved by performing comprehensive physical examinations and enhanced chest computed tomography (CT) scans. Occasionally, systemic radionuclide bone imaging was performed and, if foci of abnormal radiotracer uptake were identified, additional examinations, such as magnetic resonance imaging (MRI), CT or X-ray imaging were performed, including brain MRI or CT. In addition, B-mode ultrasound scanning of abdominal organs and the retroperitoneum were occasionally conducted and, if tissue abnormalities were detected, enhanced CT scans of the abdomen were performed. In specific cases, a whole-body positron emission tomography-computed tomography examination was conducted (4). All initial diagnoses and treatments occurred at the Tumor Hospital of Shandong Province; patients did not receive treatment from other hospitals (5). Readmitted medical cases were excluded from the present study.

### General clinical data

A total of 1,238 lung cancer patients were admitted to the Tumor Hospital of Shandong Province between January 1, 2009 and December 31, 2009. Of these 1,238 patients, 70 patients did not receive a histopathological or cytological diagnosis and had incomplete clinical data, and thus were excluded from the present study. There were 588 male smokers (71%) and 33 female smokers (9.7%) included in the study. The clinical data of the remaining 1,168 patients is indicated in Table I.

### Treatment strategies

The predominant treatment strategies received by the lung cancer patients included surgery, radiation therapy, chemotherapy, targeted therapy and comprehensive treatment involving a combination of therapies. However, certain patients refused treatment. The types of treatment were as follows: Chemotherapy alone, 164 cases (14.0%); radiation therapy alone, 28 cases (2.4%); surgery alone, 24 cases (2.1%); radiation therapy and chemotherapy, 593 cases (50.8%); radiochemotherapy and targeted therapy, 30 cases (2.6%); chemotherapy and targeted therapy, 29 cases (2.5%); radiation therapy and targeted therapy, seven cases (0.6%); surgery and chemotherapy, 116 cases (9.9%); surgery and radiochemotherapy, 58 cases (4.9%); surgery, radiochemotherapy and targeted therapy, two cases (0.2%); targeted therapy alone, eight cases (0.7%); and no treatment, 109 cases (9.3%).

### Follow-up period

As of April 30, 2012, 89 of the 1,168 patients were lost to follow-up, resulting in a follow-up rate of 92.4%. The follow-up period ranged from 1 to 41 months, and the mean and medium follow-up periods were 20.8 and 21 months, respectively. The follow-up of surviving patients is on-going.

### Statistical analysis

Statistical analysis was performed using SPSS software (version 17; SPSS, Inc., Chicago, IL, USA). Student’s t-test was utilised to compare the means of two samples and the χ^2^ test was used for all other comparisons. Univariate survival analysis was conducted using Kaplan-Meier analysis and the log-rank test, and the significance level was α=0.05.

## Results

### Patient clinical data

The clinical features of 828 and 340 male and female lung cancer patients, respectively, were compared, including age and the duration of patient delay in presenting to a doctor (Table I). The maximum and minimum ages of the male patients were 88 and 21 years, respectively; and the maximum and minimum ages of the female patients were 86 and 19 years. Furthermore, the median ages of the male and female patients were 61 and 59 years, respectively; and the mean ± standard deviation age of the male and female patients was 60.6±10.8 and 59.1±12.3 years, respectively. No significant difference was identified between the age of the male and female patients (t=1.107; P=0.271).

The maximum and minimum duration of patient delay in male and female patients was 24.0 and 0.10 months. Additionally, the median duration of patient delay in male and female patients was 2 months for both, and the mean duration of patient delay in male and female patients was 2.25±2.44 and 2.38±2.78 months, respectively. No significant difference was identified between the duration of male and female patient delay (t=0.752; P=0.816).

The predominant histopathological types of lung cancer were adenocarcinoma (n=621), squamous cell carcinoma (n=337) and small cell carcinoma (n=143). In male patients, the number of cases of adenocarcinoma, squamous cell carcinoma and small cell carcinoma were 376, 298 and 110, respectively; and in female patients, these were 245, 39 and 33, respectively. A significant difference was identified between the histopathological types of lung cancer in male and female patients (χ^2^= 8.671; P<0.001).

### Patient survival rates

As of April 30, 2012, 208 of the 1,168 lung cancer patients had received surgery alone, surgery plus radiochemotherapy and/or targeted therapy, accounting for 17.1% of the patients in the present study. The 1-, 2- and 3-year survival rates for these patients were 80.4, 44.9 and 15.8%, respectively. A comparison of the survival curves for the male and female patients in the present study indicated no significant differences.

### Association between TNM staging and patient survival

**Figure 1:**
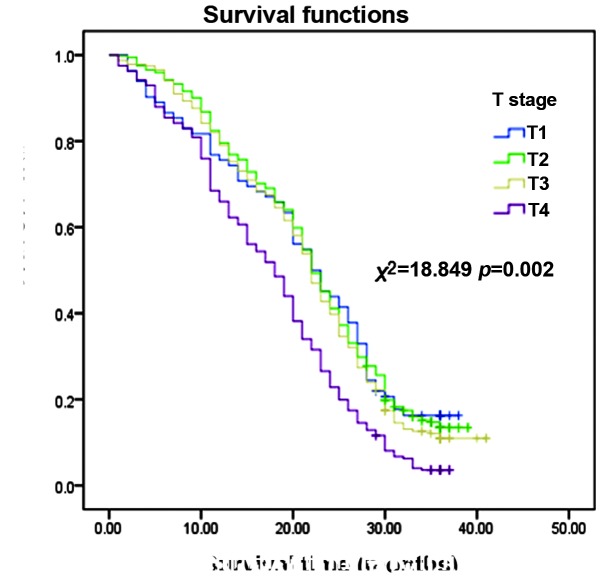
Survival curve indicating the effect of the lung cancer T stage on patient survival. T stage, extent of the tumour.

**Figure 2:**
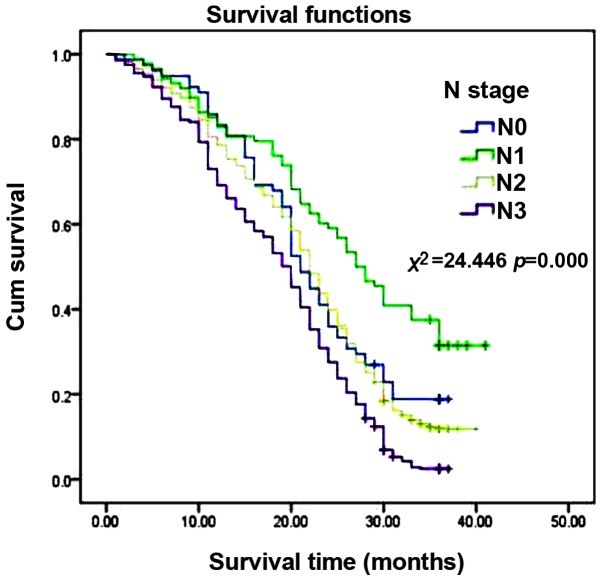
Survival curve indicating the effect of the lung cancer N stage on patient survival. N stage, extent of lymph node spread.

**Figure 3:**
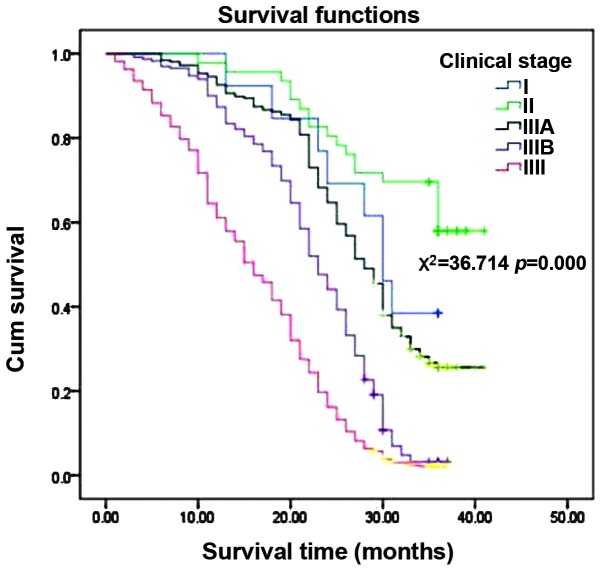
Survival curve indicating the effect of the lung cancer clinical stage on patient survival.

The effect of the tumour extent (T), lymph node involvement (N) and clinical stage of the lung cancer on patient survival are demonstrated in Figs. 1, 2 and 3, respectively. The results indicated that more advanced T, N and clinical stages of lung cancer were associated with shorter survival times.

### Association between treatment strategy and patient survival

**Figure 4:**
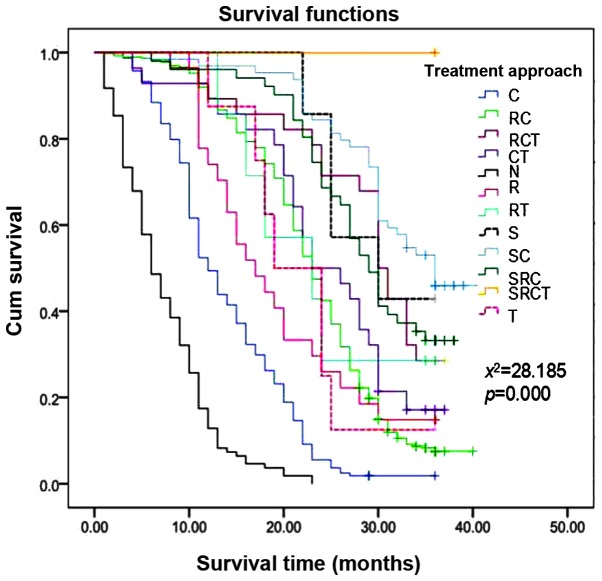
Survival curve indicating the effect of the lung cancer treatment approach on patient survival. C, chemotherapy; R, radiotherapy; T, target therapy; N, no therapy; S, surgery.

The effect of the treatment strategy on survival is demonstrated in Fig. 4. The survival curves of all of the treatment strategies demonstrated a gradual decline. Furthermore, these results indicated that a combination of surgery and chemotherapy, surgery alone, and a combination of surgery and radiochemotherapy achieved therapeutic effects. By contrast, the lung cancer patients who received no treatment or chemotherapy alone demonstrated a poor prognosis.

### Multivariate Cox regression analysis

The results of the multivariate Cox regression analysis are summarised in Table II. Patient age, T stage, N stage, overall clinical stage and treatment strategy were independent risk factors for survival.

## Discussion

The morbidity and mortality rates associated with lung cancer are the highest among malignant tumours. As medical technology advances, the diagnosis rate of lung cancer is expected to gradually rise; however, an increase in the early diagnosis rate has yet to be observed (1,2). Among the 1,168 lung cancer patients analysed in the present study, the percentages of patients exhibiting stage I, II, III and IV lung cancer were 3.9, 7.3, 42.6 and 46.1%, respectively. The percentage of patients exhibiting early-stage (stages I and II) lung cancer was relatively low, accounting for only 11.2% of the total patients analysed, whereas a significantly higher percentage of patients exhibited advanced lung cancer (stage IV; 46.1%). The percentages of stage IIIA and IIIB lung cancer patients were 23.0 and 19.6%, respectively. Under normal circumstances, surgery is not the preferred treatment strategy for patients with stage IIIB lung cancer (6). The total percentage of stage IIIB or IV lung cancer patients was 65.7%, indicating that the majority of patients were in the late stages of lung cancer at the time of the initial diagnosis and, thus, had missed the opportunity to receive surgery. It is possible that the majority of patients exhibiting early-stage lung cancer were diagnosed and treated locally, at city- and county-level hospitals, and only the patients with advanced lung cancers or complications were referred to the Tumor Hospital of Shandong Province. This may explain why the majority of patients in the current study presented in the late stages of lung cancer.

In addition, the 1,168 lung cancer patients were analysed from a gender perspective. There were 828 male and 340 female patients, resulting in a male to female ratio of 2.4:1. The incidence of lung cancer in male patients was significantly higher compared with that in females; however, no gender differences were identified with regard to the patient age at lung cancer onset or the duration of patient delay. The higher incidence of lung cancer in males may be a result of the higher prevalence of smoking among males compared with females. In the present study, a total of 588 male smokers (71%) and 33 female smokers (9.7%) were included. Smoking is a major cause of lung cancer, and numerous studies have reported a significantly worse prognosis among patients with a history of smoking compared with non-smokers (7,8). Furthermore, Chen *et al* (9) reported that smoking is positively associated with lung tumour size at diagnosis (TSD) in a dose-dependent manner. The study identified that TSD was largest in current smokers, smallest in never-smokers and intermediate in former smokers. Multivariate linear regression analysis determined that smoking status (never vs. former vs. current), histopathological tumour type (adenocarcinoma vs. squamous cell carcinoma) and gender (male vs. female) were significant predictors of TSD; therefore, gender may be a predictor of TSD due to differences in smoking duration and intensity between males and females.

In the present study, the predominant symptoms of lung cancer included cough, expectoration, blood-tinged sputum, chest pain, chest tightness and other symptoms occurring outside of the lung. The duration of patient delay ranged from 0.1 to 24.0 months, the median delay period was 2 months, and the mean delay period was 2.29 months; thus, the duration of patient delay was long. The age of onset of lung cancer ranged from 19 to 88 years, and the mean and median ages of cancer onset were 60.2 and 60 years, respectively. Furthermore, the overall cancer incidence rates gradually increased with age, and the peak incidence of lung cancer was among patients aged between 50 and 70 years. Patients <50 years of age exhibited an incidence rate of 17.7%. The results of this study similar to those of Miron et al (10), which demonstrated that NSCLC occurence was highest in the 56–71 year old age group and the most common histological type was adenocarcinoma. If any of the abovementioned symptoms are present, patients should visit a health care facility promptly and be examined by chest radiograph or low-dose CT scan to obtain a clear diagnosis as quickly as possible. In addition, it has also been reported that lung cancer screening by low-dose CT is most beneficial to asymptomatic patients ≥40 years of age. In a previous study, low-dose CT scans of 1,151 asymptomatic patients >40 years of age determined a lung cancer incidence rate of 0.51% (8/1551 patients). In addition, the incidence rates were significantly higher in high-risk patients (1.21%; 7/577 patients) and heavy smokers (2.01%; 6/298 patients) (11). The present study demonstrated that the incidence of cancer was significantly increased in individuals >50 years of age. Therefore, routine check-ups are recommended for patients who are ≥50 years of age, at least once per year, to promote the early detection, diagnosis and treatment of lung cancer.

In the current study, the predominant histopathological types of lung cancer were adenocarcinoma, squamous cell carcinoma and small cell carcinoma, which accounted for 53.2, 28.8 and 12.2% of total lung cancers, respectively. The incidence of different histopathological types of lung cancer varied between males and females: In females, adenocarcinoma, squamous cell carcinoma and small cell carcinoma occurred at a rate of 72.1, 11.5 and 9.7%, respectively; however, in males, these incidence rates were 45.4, 36.0 and 13.3%, respectively. Adenocarcinoma was the most common type of lung cancer among males and females in the present study, and the incidence rate of lung adenocarcinoma is rising in numerous countries, particularly among Asian females (12). Notably, the number of mortalities caused by lung adenocarcinoma account for 50% of the total number of mortalities caused by lung cancer-related diseases (13–15). However, Yao *et al* (16) reported that, in Western China, the incidence rates of lung squamous cell carcinoma and small cell carcinoma were increasing, whereas the incidence rate of lung adenocarcinoma was decreasing. In the United States, non-small cell lung cancer (NSCLC) accounts for almost 85% of all cases of lung cancer (17). Furthermore, discrepancies between studies may be due to the presence of regional differences in the histopathological types of lung cancer. In clinical practice, different treatment strategies should be formulated for different histopathological types of lung cancer.

Treatment strategies for lung cancer include surgery, chemotherapy, radiation therapy and targeted therapy, and different treatment strategies have different effects on patient survival. The present analysis of the association between treatment strategy and survival demonstrated that 208/1,168 (17.1%) lung cancer patients analysed were suitable candidates for surgery. The patients who were treated with a combination of surgery and chemotherapy, surgery alone or a combination of surgery and radiochemotherapy demonstrated an improved prognosis. These results indicate that survival is associated with the stage of the cancer, as surgery was generally a more feasible therapeutic option for patients with early-stage disease. The patients who received no treatment demonstrated the worst prognosis. In addition, patients who received chemotherapy or radiotherapy alone demonstrated a poor prognosis. Therefore, treatment strategies are typically associated with the general condition of the patient and the stage of the cancer. Lung cancer patients who were in poor general condition, exhibited severe chronic complications or were in the late stages of the disease received only palliative treatment or no treatment and, thus, had a poor prognosis. By contrast, patients treated with targeted therapy demonstrated an improved prognosis compared with those who received no treatment or chemotherapy alone. According to the literature, patients with epidermal growth factor receptor mutation-positive lung cancer tend to have a good prognosis following treatment with targeted therapy (9,18). However, the agents used in targeted therapy are expensive and unaffordable for the majority of patients; therefore, this treatment strategy is chosen by relatively few patients. In addition, the majority of patients who do select targeted therapy are in the late stages of the disease and have no other feasible therapeutic options available to them.

Lung cancer is a debilitating disease that results in as many mortalities as the next four most common malignancies combined, and is the leading cause of cancer-related mortality globally (19,20). The high mortality rates of NSCLC patients may be due to the fact that the disease usually presents in an advanced stage (21). In metastatic NSCLC patients, overall survival (OS) is improved by the addition of adjuvant chemotherapy treatment to best supportive care, as an initial therapy and as a therapy following relapse (22–25). Furthermore, modern chemotherapeutic approaches may be associated with positive effects on the survival of good and poor performance status (PS) patients (26).

The OS for lung cancer patients is poor (4,27). A total of 75% of lung cancer patients present with incurable advanced local or metastatic disease (28). In the present study, the 1-, 2-and 3-year survival rates were 80.4, 44.9 and 15.8%, respectively. Less than 50% of the lung cancer patients survived >2 years in the research. The main reason is late by stages. In the present study, the survival time of lung cancer patients was associated with the clinical stage of the cancer (Fig. 3). The present analysis of tumour, node, metastasis (TNM) staging and survival demonstrated that more advanced T and N stages were significantly associated with shorter survival times. Furthermore, a correlation was revealed between the overall TNM stage and the survival rate, whereas no differences were identified in the OS between males and females. Multivariate Cox regression analysis demonstrated that patient age, T stage, N stage, overall clinical stage and treatment strategy were independent risk factors for survival. In addition, patient age, T stage, N stage and overall clinical stage were negatively correlated with the survival time of the patients, whereas treatment strategy was positively correlated with prognosis. Therefore, effective treatment may improve the OS of lung cancer patients (28,29).

In conclusion, lung cancer patients typically exhibit a long duration of patient delay. The majority of patients suffer from late-stage lung cancer at the time of diagnosis, therefore, the prognosis is poor. Annual, low-dose chest CT examination is recommended for high-risk patients to enable early detection and treatment, thus improving patient prognosis.

## Figures and Tables

**Table I:** Clinical data of 1,168 patients.

	Patients	
	
Parameter	n	%
Gender		
Male	828	78.9
Female	340	29.1
Age, years		
Mean (range)	60.2 (19–88)	
<40	49	4.2
40–49	158	13.5
50–59	342	29.3
60–69	350	30.0
70–79	235	20.1
≥80	34	2.9
Smoking history		
Smoker	621	53.2
Male	588	50.3
Female	33	2.8
Non-smoker	547	46.8
Male	240	20.5
Female	307	26.3
Clinical symptoms		
Cough	87	7.4
Chest pain	74	6.3
Shortness of breath	85	7.3
Cough, expectoration	273	23.4
Cough, blood-tinged sputum	178	15.2
Cough, expectoration, chest tightness	166	14.2
Cough, expectoration, chest pain	46	3.9
Cough, expectoration, shortness of breath	64	5.5
Cough, expectoration, fever	8	0.7
Cough, expectoration, symptoms occurring outside of the lung	37	3.2
Symptoms occurring outside of the lung	102	8.7
No symptoms	48	4.1
Duration of patient delay, months		
Mean	2.29	
Medium	2.0	
Range	0.1–24.0	
Histo- or cyto-pathological type		
Adenocarcinoma	621	53.2
Squamous cell carcinoma	337	28.8
Small cell carcinoma	143	12.2
Adenosquamous carcinoma	18	1.5
Large cell carcinoma	12	1.0
Atypical carcinoid	5	0.4
Carcinoid	3	0.2
Histo- or cyto-pathological type		
Sarcomatoid carcinoma	3	0.2
Neuroendocrine carcinoma	3	0.2
Lymphoma	2	0.2
Spindle cell carcinoma	2	0.2
Other unclassified cancer	19	1.6
T stage		
T1	91	7.8
T2	582	49.8
T3	250	21.4
T4	245	21.0
N stage		
N0	125	10.7
N1	119	10.2
N2	561	48.0
N3	363	31.1
M stage		
M0	629	53.8
M1	539	46.1
Clinical TNM stage		
I	46	3.9
II	85	7.3
III	498	42.6
IV	539	46.1

T, tumour; N, node; M, metastasis.

**Table II:** Multivariate Cox regression analysis.

Factor	Regression coefficient (β)	Wald value	P-value	Relative risk
Age	−0.018	30.666	0.000	1.019
T stage	−0.140	15.427	0.000	1.150
N stage	−0.206	25.120	0.000	1.228
Clinical stage	−0.741	268.006	0.000	2.098
Treatment strategy	0.398	32.771	0.000	1.367

T stage, extent of the tumour; N stage, extent of lymph node spread.
